# Incidents of dog bites involving humans in the Khakhu Madala local area of the Thulamela sub-district, Vhembe District, Limpopo Province

**DOI:** 10.4102/safp.v68i1.6241

**Published:** 2026-07-22

**Authors:** Aifheli Rangolo, Takalani Rhoda Luhalima, Ndidzulafhi S. Raliphaswa

**Affiliations:** 1 Department of Advanced Nursing Science, Faculty of Health Sciences, University of Venda, Thohoyandou, South Africa

**Keywords:** district, human dog-bite injuries, dog owners, incident, Primary Health Care, sub-district

## Abstract

**Background:**

The separation of humans and dogs in a domestic setting may be impossible due to their long history of domestication. This close interaction can lead to conflicts and dog bites, which can cause injuries, trauma or even death. Dog bites are often underestimated, posing a significant public health risk. This study examined dog bite incidents involving humans in the Khakhu Madala area of the Thulamela sub-district in Limpopo Province, conducted within primary healthcare (PHC) facilities.

**Methods:**

A qualitative, exploratory and descriptive approach was used to investigate dog bite incidents in the Khakhu Madala local area. The study focused on registered dog-bite victims from PHC records between January 2020 and December 2021. Participants were selected through non-probability purposive sampling. Unstructured interviews conducted with 25 participants provided rich insights. Data analysis followed Tech’s eight-step criterion, ensuring trustworthiness through transferability, reliability, confirmability and credibility.

**Results:**

Key themes included a lack of owner responsibility in controlling dogs, increased dog aggression contributed to the consumption of indigenous plants and other variations and structural environmental factors.

**Conclusion:**

The study found that improving safety requires collaboration among health professionals, community engagement and evaluation of legislation. Further research on dog bite incidents is necessary.

**Contribution:**

The article highlights the prevalence of dog bites. This will assist in raising public health awareness and in informing policies and legislation to better equip them to put measures in place regarding dog bite incidents. The article also contributes to bringing education on safe dog interactions, improving treatment protocols and informing stricter enforcement of dog control laws.

## Introduction

Dog bites are a major public health issue, impacting millions annually. In the United States (US), about 4.5 million reported incidents required medical attention, with children being particularly at risk.^1^ Dog bites are the 13th leading cause of nonfatal visits to emergency departments.^2^ Similarly, studies^3,4^ have reported millions of injuries worldwide, mainly affecting children. Males account for over 50% of animal-related injuries among dog bite victims. Dog bites can range from minor punctures to severe wounds, especially on the arms, face, scalp, neck and hands. Overall, dog bite incidents seem to be decreasing; however, some breeds – especially among stray animals – are still prone to causing serious attacks.^5^

The research highlights that dog bites remain a significant global public health issue, causing injuries that range from minor to severe.^6^ Forensic pathologists have investigated serious cases, assessing injuries and dog characteristics. Male dogs, generally more aggressive than females, account for about 75% of incidents. While dog bite fatalities are rare, 468 deaths have been reported in various countries, such as in the US, from 2011 to 2021.^7^ To reduce incidents, the US implemented strategies that included public education on responsible pet ownership, stricter leash laws and breed-specific legislation. Research from low-and middle-income countries, such as South Africa, showed that about 72.3% of dog bites are from owned dogs.^8^ In addition, another study showed that dog bites are 87.2% more frequent than other forms of animal aggression.^9^ This makes dog bites a significant public health concern.^10^ In South Africa, dog bite incidents were especially high among children, with domestic dogs responsible for a staggering 81% of 98 743 reported animal bites in Limpopo Province from 2011 to 2023. In addition, the risk in workplaces has also increased because of aggressive breeds such as Boerboels. It should also be kept in mind that rabies remains a serious issue, leading to 32 fatalities in the Vhembe District, highlighting the need for mass vaccination and improved medical access.^11^

There is no known research on factors contributing to rising dog bite incidents in the Khakhu Madala area of the Vhembe District. Importantly, South Africa – including this particular community – needs both better awareness of dog-keeping laws and the engagement of traditional leaders to help establish bylaws to reduce dog bites.

This study examined dog bite incidents in the Khakhu Madala area of the Thulamela sub-district in Limpopo Province. Dog bites remain common, but society often reacts to incidents rather than focusing on prevention.

### Problem statement

The Khakhu Madala local area in Vhembe District, Limpopo Province, was experiencing a rise in human-dog bite incidents, with a weekly rate of 10 incidents. In 2017, 129 dog bite cases were reported from Primary Health Care (PHC) facilities, increasing to 156 in 2018. The community primarily consists of middle-aged males and females aged 17–30 years, with some minors and elders affected. Therefore, investigating the factors contributing to these incidents was crucial.

### Rationale of the study

Dog biting has been a persistent health issue, yet society often treats it reactively. This research aimed to address this issue and contribute to the World Health Organization (WHO) 2030 Vision of zero human rabies deaths. Factors contributing to dog bite incidents vary across settings and communities. In the Khakhu Madala community, it appeared there was a knowledge gap on dog-keeping laws. Encouraging awareness among influential individuals, such as traditional leaders, can help reduce dog bite incidents.

### Purpose of the study

This study explored dog bite incidents in the Khakhu Madala area of the Thulamela sub-district in Limpopo Province.

### Objectives of the study

To determine the factors contributing to dog bite incidents in the Khakhu Madala local area in the Thulamela sub-district of Vhembe District, Limpopo Province.

## Research methodology and design

### Study design

The study utilised a qualitative, explorative, descriptive and contextual design to explore human dog bite incidents in the area. It employed unstructured interviews to investigate the causes of these incidents.

### Study setting

The study focused on the Khakhu Madala local area in the Thulamela sub-district of the Vhembe District, Limpopo Province. The area is characterised by mountainous terrain, poor road infrastructure and a lack of public transport, affecting access to healthcare services. The community is commonly known for owning domestic animals. Socioeconomic challenges in the area include high unemployment rates, rising crime and crop destruction. Government services are distant, and there are no agricultural offices, animal clinics or public transport. The population is mainly made up of middle-aged males and females aged 17–30 years, with few minors and elders, and a culture of sharing and increased social contact. There are seven PHC facilities – apart from each other – which appear to be limited to a wide area of settlement.

### Study population, sampling and sample size

This study examined dog bite victims recorded in the dog bite register at the Khakhu Madala local health facilities from January 2020 to December 2021. Participants were selected by their traceability, with the register providing their addresses, contact numbers, bite locations, injury severity and management plans, which facilitated interviews at their preferred PHC facilities.

Using non-probability purposive sampling, the researcher chose individuals who were willing and able to share valuable insights. The inclusion criteria were dog bite victims recorded in the dog bite register of Khakhu Madala Local Area PHC facilities between January 2020 and December 2021. The researcher objectively selected from this pool of participants who were accessible, able, willing and knowledgeable about the dog bite incidents that had occurred. Dog bite victims under 18 years of age were excluded. Data saturation was achieved at participant 20, when no new information emerged.

### Recruitment of the participants

The University of Venda (UNIVEN) Human and Clinical Trials Research Ethics Committee approved a research project (number SHS/21/PDC/10/1908). Authorisation was secured from PHC facilities and community leaders, including the head of the Limpopo Department of Health and the Traditional Council. Participants signed an informed consent form, which included a letter detailing the study’s title, purpose, procedures, risks, benefits, withdrawal conditions, interview duration, remuneration and confidentiality.

### Data collection

The author started data collection following authorisation from relevant authorities. A pre-test of the central question was conducted with two participants, which was not included in the main study, and their results were also excluded from the analysis. Participants were recruited by phone the day before the interviews, during which they were informed about the study’s objectives and provided with written consent. In line with the methodology used by Gobbo and Šemrov,^12^ unstructured in-depth interviews were conducted in the Tshivenda language. A language specialist fluent in Tshivenda translated these interviews verbatim into English. The central question posed during the interviews was: ‘What do you think are the contributing factors to human dog bite incidents in the Khakhu Madala local area?’ It was then followed by several probing questions to get in-depth information.

This study used unstructured in-depth interviews with adult patients at PHC facilities to investigate factors contributing to dog bite incidents. The researcher maintained neutrality, active listening and empathy, and referred participants to social workers if needed. The author conducted the interviews, which were audio-recorded during participants’ lunch breaks, each lasting 35–45 min. Data collection was done from July to September 2022. The study sampled (*N* = 25) dog bite victims registered at local health facilities between January 2018 and December 2019, focusing on individuals aged 18 years and older. Data saturation occurred after 20 interviews, with three more added to enrich the findings. The majority were male participants aged 18–34 years, with fewer female participants in the older age group. This data provided valuable insights into the demographics and circumstances surrounding dog bite incidents.

### Demographic summary

Interviews were conducted with 25 participants. Compared to female participants, male participants were more with a difference of 7. Among the male participants, nine were between the ages of 18–35 years, while seven were above the age of 35 years, resulting in a total of 16 male participants. On the other hand, there were three female participants aged 18–35 years and six female participants aged 35 years or older, bringing the total number of female participants to 9. Thus, a total of 25 participants – including both males and females – were included in the study.

### Data analysis

Data were analysed according to Tesch’s open-coding steps, as indicated by Reese and Vertalka.^13^ Data analysis was achieved by reading data and transcribing it to get a sense of the whole narrative before breaking it into parts. Table 1 presents the summary of themes and sub-themes of the study.

**TABLE 1:** Summary of themes and sub-themes.

Themes	Sub-themes
**Theme 1:** Lack of responsibility in dog control by dog owners	1.1. Negligence 1.2. Inadequate practice in dog tethering 1.3. Lack of dog cages 1.4. Owning a dangerous dog breed
**Theme 2:** Increased dog aggression linked to consumption of indigenous plants and other variations	2.1. Animal cruelty
**Theme 3:** Structural and environmental factors	3.1. Lack of veterinary services 3.2. Free-roaming sick dogs

### Ethical considerations

Ethical clearance to conduct this study was obtained from the University of Venda and the Human and Clinical Research Ethics Committee. The ethical clearance number is SHS/21/ PDC/10/1908.

## Results

The study identified three themes: lack of responsibility in dog control by dog owners, increased dogs’ aggression linked to consumption of indigenous plants, and structural and environmental factors. Sub-themes emerged during interviews, with interpretations set out in Table 1.

### Theme 1: Lack of responsibility in dog control by dog owners

Dog tethering is a method of restraining a dog to a stationary object, such as a tree, in the pet owner’s yard. Factors that stimulated dog bites included confining dogs in small spaces, restricting them to dangerous areas and keeping them confined for long hours without basic needs. Four sub-themes emerged: negligence, inadequate dog tethering practice, lack of dog cages and owning a dangerous dog breed. This was evidenced by the participants who said:

‘The dog owner chose to tether his dog in the wrong site, which is near the passage route that community members frequently pass by, which exacerbates the dog’s aggression frequently and compromises the quality of the rope to fray and break easily, resulting in a dog bite. Furthermore, this lack of responsibility in the control of the dog by the owner clearly shows that the dog owner inadequately practices dog tethering. It should be borne in mind that restricting a dog within a site where everybody passes by, including young girls and boys, may provoke the dog when they pass by and pose a serious risk.’ (Participant L)

‘The dog owner brought his dog to the funeral, trying to create an opportunity for his dog to feed itself on the leftovers. This clearly shows that there is also a lack of responsibility in control of dogs by the dog owner and failure to feed the dog as he relied on garbage that remains during events, which is not the right food for a domestic dog.’ (Participant F)

Dog owners persistently restrict their dogs, causing stress and exposing them to extreme temperatures. This led to stress, fights and fraying of the leash, making the dogs vulnerable to aggression and causing them to bite.

#### Sub-theme 1.1: Negligence

Negligence arises from inadequate requirements such as food, shelter, a healthcare plan, tethers, timing, sight and communication for dog owners. Dog owners should possess these requirements and be held responsible for their dogs as illustrated by the following statements:

‘The dog escaped the yard in the morning in the presence of the dog owner, and the dog didn’t heed its owner’s call, but what hurts most was the lack of action to take control over the dog as an owner who chose to go to work before the dog was kept under control. This resulted in the dog going astray and not caring about the safety of the people the dog may encounter in the street. This kind of negligence subjected me to the dog bite incident as the dog chose to go and lie at my household gate. This incident could have been prevented, but the dog owner didn’t take advantage of the opportunity and report to the nearest veterinary services for assistance.’ (Participant L)

This showed that the dog owner was negligent by failing to provide the basic needs for his dog and inadequately practising keeping his dog under control for extended hours. In addition, the dog owner lacked responsibility for whom to consult for assistance when needed. The following quotations supported this view:

‘I felt so disturbed when the dog owner neglected his dog, as he failed to provide the basic needs for his dog. Bringing a dog in public and allowing it to feed on leftovers is evidence on its own that the dog owner has neglected their own dog because they do not provide care for their own dog. Most dog owners, when going in public spaces, put dog collars in order to control the movement of their dogs so that they do not easily break into a fight, and they do not roam around.’ (Participant T)

‘After the dog bit me, the dog owner was not ready to own the responsibility, as they were pretending that they were not the dog owner. It became clear that the dog is theirs when people beat it up, and the dog shielded around him for safety.’ (Participant P)

Participants expressed that dog owners needed to understand the caring and handling of dogs, not tether them where they could be easily provoked by people passing by.

#### Sub-theme 1.2: Inadequate practice in dog tethering

The participants revealed that dog owners failed to provide adequate care for their dogs as they broke the tether and went astray searching for food. The participants asserted that incidents were influenced by dog owners restraining their dogs with inappropriate material for tethering and failing to provide the dogs with basic needs such as water and food, prompting them to break the tether to search for food. The following quotes affirm this:

‘The incident happens because of a lack of adequacy in the practice of dog tethering. The dog owner used to restrict the dog’s movement using breakable materials and even forgets to change the positioning in different weather. The incident of a dog bite happens as a result of the dog having broken the tether and escaped through a broken fence. Unknowingly, I came into an encounter with a dog in the street as it was searching for food. The dog bit me as I was performing gestures to scare the dog so that it would not come closer to me. The dog resisted and then responded by biting me up, defending its meal. Although the dog had a broken piece of tether around its neck after being abandoned in hot weather, it fought and succeeded in breaking the tether. It was also evident that the material for tethering was not of quality.’ (Participant M)

‘Naturally, dogs are territorial animals and restricting them to a small space causes them to respond aggressively and territorially to the approach of people and manifest their emotions by barking or biting. These dog owners are negligent and practice dog tethering inadequately, as they put their interests first over the general people’s interests by creating no-go areas in public spaces. We live in fear, even in a public space, because the situation is unpredictable. Dog owners sometimes are inconsistent with their tethering practice, as you may find a dog not restricted, aggressive, or dangerous, as it may be.’ (Participant A)

‘While chaining does not always make a dog aggressive, the animal is being given fewer options in fighting circumstances, thus inviting situations that increase the likelihood of aggressive responses. Chained dogs are time bombs and a risk to public health, especially those of dangerous breeds, which are not even fit to be kept in a domestic environment.’ (Participant D)

Participants explained that dogs become aggressive and bite community members because of neglect by owners who fail to provide basic requirements. The materials used to restrict dogs, such as ropes, chains, wires and cables, can fray and break easily, allowing dogs to run loose and bite people. In addition, prolonged dog tethering without opportunity for free movement increases the risk of bite incidents.

#### Sub-theme 1.3: Lack of cages

Dog bite incidents are largely because of a lack of cages. This results in deviations from dog restriction principles, such as unsecured yards, broken fences, frayed materials and inconsistencies in tethering dogs. Dog owners often fail to maintain their fences, and some do not even have a fence at all. This is supported by the following participant statements:

‘Unknowingly, as I was visiting my uncle, I was travelling along the street, which led me to my uncle’s household. The street was very quiet and made me wonder why there were no people from the opposite direction or even behind me. After moving a few meters ahead, a pack of three dogs escaped the yard and started to attack me. With the bag that was in my hand, I tried to protect myself, but it was not enough. I fell on the ground screaming for help; luckily, a car arrived, hooting to scare the dogs. One passenger opened the door and grabbed me on my jacket for safety. However, the dogs were still aggressive as we drove off the scene for safety. This was horrible for me as I lost a part of my left ear, bleeding heavily. Some of the local community members gave their expressions about the uncooperative behaviour of the dog owner, who is reluctant to keep his dogs under control, as he was advised to keep them in a cage for the safety of people. Other informants reiterated that the dog owner lets his dogs lose as he is unable to feed them. This means that the dog owner has been negligent by lacking a dog cage to keep his dog under control, and even inadequately practising his dog tethering in this regard. This ultimately led to me being bitten.’ (Participant V)

‘I was so disturbed by how the dog owner had hugely contributed to this dog bite incident. This is because he has not been consistent with tethering practices, though he is aware that customers frequently visit his home to buy some grocery items. The dog owner didn’t have a cage to keep his dog and used tethering materials that fray. In this regard, tethering was inadequately practised, and he should have a dog cage as an alternative for dog control.’ (Participant A)

According to the participants in this study, the fault lies with dog owners for not having dog cages and compromising other people’s safety.

#### Sub-theme 1.4: Owning a dangerous dog breed

This study found that participants believe dog owners often keep dangerous breeds, such as German Shepherds, known for their muscular build and aloofness. They also shared a frightening incident involving an aggressive Pitbull. The study highlights the risks of having dangerous dog breeds in households. They claimed that dog owners keep dangerous breeds as pets, such as German Shepherds, which are characterised by their raised ears, well-built muscles, fearlessness and aloofness. They also mentioned a horror attack by a Pitbull, which was aggravated by the dog’s aggressiveness and potential bite parts. The study highlighted the risks of keeping dangerous dog breeds in households.

The following quotes indicate the participants’ responses:

‘I was bitten while protecting my small dog as I was about to leave the restaurant in the afternoon. The dog owner of a Pitbull found me seated, holding my small dog with a harness on their arrival at the restaurant. The dog then became restless, though it was under the control of the harness. It pulled the owner, forcing them towards me as it tried to approach my small dog. Eventually, the Pitbull owner’s harness slipped out of their hand, and the Pitbull was all over my small dog. When my dog tried to hide between my legs, and as I tried to break up the fight, the Pitbull then intensified the fight such that I was also bitten multiple times on my body. The dog was tearing everything apart as the aggression heightened, and my dog and I were now victims. When the security calmed down the fight, the Pitbull was still eager enough to continue the fight. From what I have gone through and observed about this phenomenon, the Pitbull owner was just keeping a dangerous dog. They are even unable to calm it down when it is aggressive.’ (Participant R)

‘Unknowingly, I opened the household gate to recollect the cellular phone that I forgot as I left my part-time job. I was terrified of coming across a very big dog, which I didn’t have the guts to stand and defend myself from. As I chose to run away for my life, it ultimately caught me and broke my left hand with only one bite. I really thank the dog owner who spotted the incident and responded very quickly, considering its huge and brave physical appearance, the character of a Pitbull.’ (Participant D)

The sub-theme of owning dangerous dog breeds provided critical insights. Participants showed that there are specific dog breeds that have been revealed to be dangerous and can pose a danger to people in the community.

### Theme 2: Increased dog aggression linked to consumption of indigenous plants and other variations

#### Sub-theme 2.1: Animal cruelty

Participants expressed that dog bites are often attributed to the use of indigenous plant products, such as Mulanga, which heightens aggression in dogs. This practice involves crushing the leaves of the plant, forcing the dog to sniff to deliberately exacerbate its aggression. Farmers in the Khakhu Madala area often introduce Mulanga to their dogs during summer to guard against wild animals and humans. This practice increases dog bite incidents, but seasonal changes can also affect dog behaviour. Participants also shared their experiences of aggression augmentation, where the owner and the substance provider failed to adhere to their agreement. The practice is seen as a way to control dog aggression. This is supported by the following quotes:

‘I learned to know the incident of my dog bite following arguments between the provider of the substance used to augment aggression in dogs and the dog owner when it ensued, as it was mentioned that the dog owners owed a certain amount of money for the service rendered on their dogs to beef up security at home following theft. The behaviour of the dog was strange following the influence of the indigenous plant, which was introduced for its aggression. This kind of practice sounds inhumane as it risks innocent people’s lives.’ (Participant J)

In another incident, a participant that was a victim, added:

‘I was bitten multiple times outside the crop farm by the dogs that trespassed the broken yard. The incident happened as I was going to buy vegetables in a nearby crop farm. The dog owner was alleged to have introduced the indigenous plant to his dogs for them to become aggressive. In other words, he is leaving his dogs on the farm as guards against crop theft and destruction by wild animals during his absence; this is found to be disturbing because the fence of the farm was broken, which makes it easier for the dogs to attack people who used to pass by.’ (Participant Y)

The previous statement provided evidence of deliberately heightened dog aggression. Although the influence of the summer season cannot be examined in isolation from other overlapping factors contributing to the dog bite incidents.

### Theme 3: Structural and environmental factors

The study revealed structural and environmental factors contributing to dog bites in Khakhu Madala, a rural area in Vhembe District. Primarily, the absence of local veterinary services and the presence of infected free-roaming dogs enhance the community’s lack of access to veterinary care. Because of the area’s remoteness, existing animal care services are centralised, making it difficult for community members to access them. Furthermore, traditional leaders lack expert knowledge and rely on community members for resolution, making the situation unjustifiable. One of the participants opined:

‘This has been one of the most horrific attacks I have ever experienced following an encounter with a pack of three dogs that grievously bit me. Though the other two dogs were strays, the third dog was neglected by its owner, which raised the traveling and medical costs for the dog, as it was sick. The dog owner, in this regard, left his dog in an abandoned place following the dog’s sickness. The other dogs were unknown, as there was an increased number of stray dogs in the communities around. Looking at this incident, it is clear that, as rural communities, we are left in the dark regarding animal care services that are centralised in urban areas by the Department of Agriculture and Forestry. If these services were not centralised, dogs would not be roaming around unaccounted for.’ (Participant C)

The participant raised concerns about the owner’s inaction on sick dogs and unknown dogs roaming, indicating a higher vulnerability to dog bites because of a lack of veterinary services.

#### Sub-theme 3.1: Lack of veterinary services

The study revealed that the lack of veterinary services and regular vaccination campaigns in addition to a lack of information on addressing stray and rabies-symptomatic dogs contributed to the bites. It is difficult to identify a single animal service in communities; most facilities with animal expertise are centralised, making them difficult to reach. Community members often kill these dogs without seeking expert advice, following a lack of information on how the dog can be treated. One of the participants responded by saying:

‘The dog owners turn to abandoning their dogs when they are sick due to a lack of facilities for animal clinics, such as veterinary services or the Department of Agriculture and Forestry. They dump their dogs far from home when they do not want to take care of their sick dogs, and they end up biting people. I once found myself in a dire situation when a dog owner rejected the claims after his dog was involved in a bite incident, even though he failed to take it to the animal clinic. It was evident by the look in the dog’s eyes that the dog was sick, and some community members even wanted to kill the dog for fear of spreading the disease to the people and other dogs around. Because of the lack of expert intervention in this regard, people tend to participate in some form of cruelty when they influence each other to kill those dogs to resolve the problems. This decision is taken without expert advice pending veterinary service in rural communities, as the services are centralised in urban settings.’ (Participant G)

#### Sub-theme 3.2: Free-roaming sick dogs

Free-roaming dogs are highly susceptible to infectious diseases like rabies, posing a significant risk to both animal welfare and public health:

‘Coming home from work is no longer safe, as many dogs are roaming up and down in the households and even in the street. I was bitten by a dog roaming in the street as I was leaving work. The dog had been stalking me for a distance as I was carrying junk food in my bag. I chased the dog at first, and it ran away; how it returned was a surprise, as it bit me on the upper leg on the side where I was carrying the food. Two other dogs were approaching from the side, and when they noticed the approach of the other two people towards me at a distance, they fled to the nearby bush. The density of dogs has increased in our community, which is a serious problem for the community. The stakeholders responsible in the Departments are not heeding the call, as the community has been raising voices about this problem. This confirms that the structural environment regarding animal control does not exist.’ (Participant U)

Another participant added:

‘Until today, I don’t even know the owner of the dogs that were destroying our livestock. This is because I fell victim to a dog bite as I was protecting our livestock on a farm when we discovered that dogs were coming at night to attack our livestock. As I was one of the community members who mobilised to attack those dogs at night, it happened that the dogs also fought back to escape the cage in which they were trapped. This is when the dog aggressively turned against me and bit me badly, such that I was also referred for major stitches at the hospital.’ (Participant H)

The study showed that factors contributing to dog bite incidents include animal cruelty by owners, community influences, a lack of awareness by animal care experts and centralised veterinary services.

## Discussion of themes and sub-themes

The following discussion will be based on the developed themes.

Dog tethering refers to the practice of restraining a dog to a stationary object, such as a tree, to maintain control.^14^ Participants mentioned dog tethering in incidents, indicating owners disregard the pain and suffering caused by bitten community members. Dog owners disregard tethering site locations, restraint duration, materials, and shade availability. These behaviours lead to several sub-themes identified in this study.

To establish safety from either side, namely, the dogs and people around, the dog owner must abide by certain principles prescribed by the Royal Society for the Prevention of Cruelty to Animals (RSPCA),^15^ which prescribes the following: training of the dogs on tethering, the use of an appropriate tether design, consideration of the tethering site and timing and duration of tethering.^16^

To clarify the principles,^17^ it was emphasised that the tethering of dogs must comply with any state and territory laws or regulations relating to the tethering of dogs. Furthermore, provisions should be made that the tether design should be swivel, made of leather, approximately 3 m long, since it reduces the likelihood of entanglement. It was also added that metal chains are preferable as they provide greater security than a rope, since they do not fray.

The study conducted by the 353 RSPCA^18^ elaborated on the tether sites, which must be reasonably flat, dry, non-obstructive, away from footpaths, and under shade during hot weather. In addition,^18^ it alluded to the conditions that warranted avoiding tethering and mentioned long-term, extremely cold and hot weather, nursing or pregnant bitches, or near whelping.

The study conducted by^19^ RSPCA alluded that dogs are territorial animals that respond aggressively by barking and biting when restricted for long hours. Furthermore,^19^ RSPCA identified various types of behaviours and circumstances that can lead to dog bites. These include defensive behaviour, distancing, territorial aggression, protection of materials, irritability, fearfulness, displacement, competition between dogs, competition between dogs and people, hunting and responding to commands. The study conducted by Elliott et al.^20^ indicated that the RSPCA emphasises the importance of safe tethering, recommending that it should always be done away from footpaths and roadways for everyone’s safety. Furthermore, it concluded that dog tethering practices were highly inadequate, as dog owners failed to meet even one of the above-mentioned requirements.

According to earlier conducted research,^21,22^ certain aspects have been prescribed that must be adhered to when tethering a dog. Furthermore, the previous research^23^ has also mentioned circumstances that do not require tethering, such as long-term, nursing or pregnant bitches, or near whelping and during extreme hot or cold weather.

Researchers^24^ have also emphasised that dogs should not be tethered for long periods. In the Khakhu Madala area, however, dogs were often tethered indefinitely until their ties frayed or broke, mainly because of a lack of cages. Participants noted that dog owners sometimes contributed to bite incidents because they did not have cages, leading to dogs escaping through broken fences and roaming the streets. Furthermore, it was mentioned that abandoned dogs increase the stray dog population. Moreover, free-roaming sick dogs contribute to an increase in incidences of dog bites, as they lack appropriate care from their owners.

A study in Harris County, Texas, found that the Pitbull breed had the highest bite frequency compared to Labrador Retrievers, although it was not deemed dangerous.^25^ Despite their genetic traits leading to greater restrictions, incidents involving Pitbulls contribute to the perception that they are dangerous. This belief is reinforced by reported incidents, such as the tragic case on 13 November 2022, where a Pitbull bit an 8-year-old boy in Vista Park, Free State Province.

In another study conducted in Liverpool, some dog bites were regarded as play bites and were perceived as normal if they did not involve a Pitbull. Moreover, research^26^ also found that dog bite incidents only become a concern if a Pitbull is involved. The rationale behind this was that Pitbulls are notoriously vicious and hateful, such that they attack people until they maul their prey and are proud of what they accomplish. Based on earlier research,^27^ it can be concluded that Pitbulls are more dangerous than other breeds.

However, following the above-stated information, the researcher did not know how many dog breeds existed within the Khakhu Madala study population. Thus, he cannot say whether the Pitbull breed is dangerous. The perception that the researcher got was that a Pitbull was involved in most cases by visual identification. The pictures taken from different incidents of dog bites had different breeds, but most of the bite characteristics were of a Pitbull compared to other breeds.

### Limitations of the study

The study focused on factors contributing to dog bite incidents in the Khakhu Madala local area of Vhembe District, Limpopo Province.

### Strategies to ensure trustworthiness

Trustworthiness is crucial for ensuring the validity and reliability of research findings regarding dog bite incidents involving humans in the Khakhu Madala area of the Thulamela sub-district.

To establish trustworthiness, the accuracy of findings was ensured by thoroughly engaging in the research setting. This promoted an in-depth understanding of the phenomenon and involved triangulation through interviews, observations and diary entries. Trustworthiness in research is a subjective concept, with varied findings arising from different researchers, locations and types of writing courses. Qualitative researchers establish trustworthiness through four criteria: credibility, transferability, dependability and confirmability.

Credibility refers to the degree to which readers believe the researcher has accurately captured participants’ perspectives. Transferability pertains to the ability to apply the findings to other contexts or participant groups, particularly regarding incidents of dog bites involving humans in the Khakhu Madala local area of the Thulamela sub-district.

Dependability assesses whether the findings can be replicated, which is achieved by learning from participants without exerting control over them. This was accomplished through inquiry audits and stepwise replication techniques.

Confirmability aims to remove biases from research processes and results, ensuring that findings, conclusions and recommendations are based on the data. This was achieved through inquiry audits of raw data, vigilance against preconceived notions and ongoing engagement between the researcher and their supervisor.

### Recommendations

Healthcare workers should raise community awareness about dog bites by collaborating with regulatory bodies and non-governmental organisations. They should inform communities about rabies fatalities and precautionary measures. Dog owners should avoid aggression, and policies should require licensing for defined periods. Decentralisation of animal wellness services to rural communities, awareness of the WHO’s Vision 2030 target and accessibility of animal care services should be prioritised. Royal councils should formulate bylaws for vulnerable communities.

## Conclusion

The findings from this study revealed that dog owners often neglect their dogs’ safety by tethering them to stationary objects, leading to incidents and potential negligence. The RSPCA recommends training dog owners on tethering, using appropriate designs and complying with state/territorial laws. However, dog owners often fail to meet these requirements, leading to territorial and aggressive behaviour by their dogs. In the Khakhu Madala local area, dogs were tethered for an undefined period, leading to dog bite incidents, particularly involving Pitbulls. The study aimed to educate veterinarians on dog morbidity outbreaks and interventions, alleviate the burden on the National Department of Health and contribute to the knowledge on dog bites.
